# Effect of Red Wine Intake on Serum and Salivary Melatonin Levels: A Randomized, Placebo-Controlled Clinical Trial

**DOI:** 10.3390/molecules23102474

**Published:** 2018-09-27

**Authors:** Elena M. Varoni, Rita Paroni, Jacopo Antognetti, Giovanni Lodi, Andrea Sardella, Antonio Carrassi, Marcello Iriti

**Affiliations:** 1 Department of Biomedical, Surgical and Dental Sciences, via Beldiletto 1/3, Università degli Studi di Milano, 20142 Milan, Italy; elena.varoni@unimi.it (E.M.V.); giovanni.lodi@unimi.it (G.L.); andrea.sardella@unimi.it (A.S.); antonio.carrassi@unimi.it (A.C.); 2 Department of Health Sciences, via Di Rudinì 8, Università degli Studi di Milano, 20142 Milan, Italy; rita.paroni@unimi.it (R.P.); jacopo.antognetti@gmail.com (J.A.); 3 Department of Agricultural and Environmental Sciences, via G. Celoria 2, Università degli Studi di Milano, 20133 Milan, Italy

**Keywords:** Mediterranean diet, indoleamines, nutraceuticals, functional foods

## Abstract

Melatonin (MLT) is a recently discovered phytochemical in wine, but its influence on physiological MLT levels is still unknown. This study aimed at evaluating variations, in serum and saliva, of MLT concentrations after the intake of MLT-enriched red wine. Twelve healthy volunteers were recruited to receive 125 mL of red wine naturally lacking of MLT (placebo, PLC), or the same wine enriched with MLT (MLT+). A physiological steady decline of serum MLT was observed from baseline up to 90 min, for both wines. After PLC intake, the decrease was significantly faster than the one occurring after MLT+ wine, which thus delayed the drop down of serum MLT with a plateau at 30–60 min. Salivary MLT levels slightly peaked at 45 min after MLT+ wine intake, without statistical significance. Therefore, the intake of a glass of MLT-enriched red wine changed serum levels of the indoleamine, supporting the role of wine MLT in counteracting the physiological decline of the hormone into the bloodstream.

## 1. Introduction

Although the paradigm of red wine health-promoting effects has been largely attributed to polyphenols, in the last decade, a new molecule enriched this scenario: melatonin (MLT) [1]. Dietary MLT may, indeed, have direct beneficial effects and maximize the biological activities of micronutrients (vitamins and minerals) and other phytochemicals present in plant foods [2,3].

Mercolini and colleagues [4,5] found 0.5 ng mL^−1^ of MLT in Sangiovese red wine, and 0.6 and 0.4 ng mL^−1^ in Albana and Trebbiano white wines, respectively. Stege and co-workers reported MLT at 0.16, 0.24 and 0.32 ng mL^−1^ in Chardonnay, Malbec and Cabernet Sauvignon wines, respectively [6]. Results from our group showed the levels of MLT in Groppello and Merlot wines varied between 5.2 and 8.1 ng mL^−1^, depending on the agrochemical treatments [7]. Other authors reported even much higher MLT concentrations, up to 130 and 420 ng mL^−1^, in red and white wines (Cabernet Sauvignon, Merlot, Syrah, Tempranillo, Tintilla de Rota, Petit Verdot, Prieto Picudo and Palomino Fino) [8]. Again, in a screening of Italian mono- and polyvarietal red, white and dessert wines from different geographical areas, we measured MLT at concentrations around or less than 0.5 ng mL^−1^ [9]. However, the presence of this indoleamine in wine is not always the rule: MLT in grape (cv. Malbec) berry skins was detected within the range 120–160 ng g^−1^, but it was no more present in the experimental wines (produced from the same grapes); here, a MLT isomer was identified, in the range 18–24 ng mL^−1^ [10]. In another study, MLT was found in berry skin (440 ng g^−1^), but not in the experimental and commercial wines, whereas a MLT isomer was measured in wine (from 60 to 211 ng mL^−1^), but not in berry skins [11]. This great and unpredictable variability of results is likely due to factors that may influence the biosynthesis of MLT in grapes and, consequently, the levels of this indoleamine in wine, as well as factors related to vinification. Among the others, genetic traits of cultivars, phenological stages of development, agrochemical treatments, climatic and agro-meteorological conditions and winemaking procedures could play a pivotal role [12].

To complicate the picture further, oral bioavailability may affect the health-promoting effects of dietary phytochemicals [13], including MLT, as it relies on the biotransformation system of the individual, on the chemical structure of each compound, as well as on the high complexity of the food matrix, as the red wine actually is.

Although oral availability of red wine polyphenols has been investigated [14,15], no information is currently available on oral bioavailability of MLT after drinking a glass of red wine containing this indoleamine. Noteworthy, differently from polyphenols, which are plant-exclusive metabolites reliably detectable in humans, the levels of exogenous MLT coming from foods (usually in the order of ng/g or ng/mL) cannot be distinguished unfailingly from the levels of MLT endogenously produced. Indeed, there is still a lack of knowledge about oral bioavailability of dietary MLT from red wine, a complex food matrix, though its pharmacokinetics as dietary supplement (at much higher doses) in forms of capsules, spray or gel has been known for decades [16,17]. The complexity of food matrix, in turn, could strongly influence absorption of bioactive metabolites, including MLT, present in foods, thus affecting the biological significance of the molecule.

This study aimed at investigating, for the first time, the levels of MLT in serum and saliva soon after the intake of a MLT-enriched red wine.

## 2. Results

**Figure 1:**
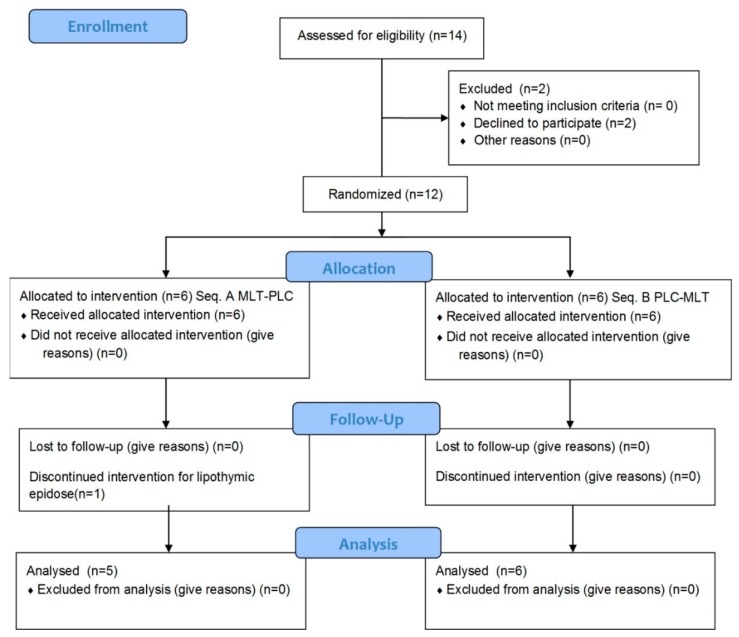
CONSORT (consolidated standards of reporting trials) flow chart of the study design.

Fourteen individuals were contacted, but two of them refused for personal reasons (no wine drinking) (Figure 1).

Thus, twelve volunteers were recruited (seven males and five females; mean age: 25.8 ± 5.9 years; mean BMI: 21.3 ± 2.5 kg m^−2^). Demographic and anthropometric characteristics of each volunteer and dietary habits are detailed in Table 1. Eleven out of twelve volunteers completed the study, with high compliance. Only female participant No. 10 dropped out because of a lipothymic episode during the blood withdrawal at baseline.

### 2.1. Melatonin Concentrations in Serum and Saliva

**Figure 2:**
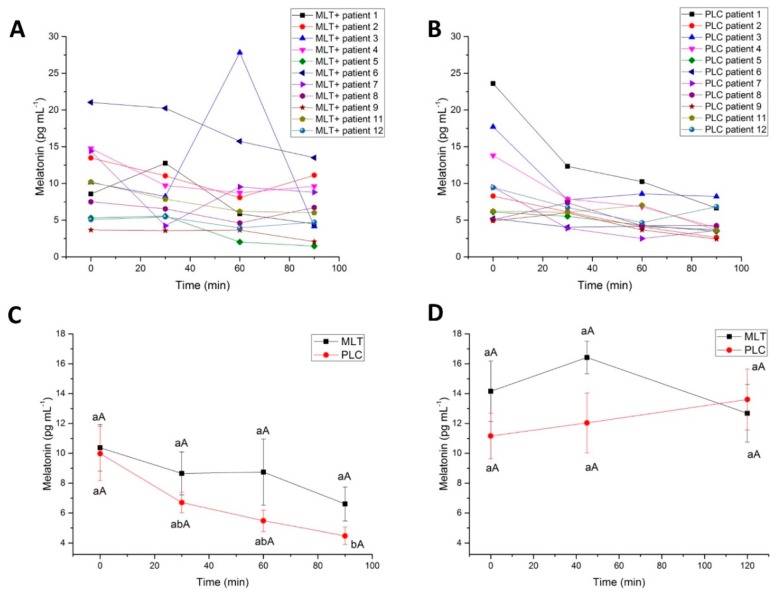
(**A**,**B**) Time-course of the serum melatonin levels in each of the healthy volunteers from 0 min to 90 min after administration of red wine (**A**) without melatonin (placebo, PLC) or (**B**) the same wine fortified with melatonin (MLT). (**C**) Time-course of the serum melatonin mean levels in healthy volunteers (*n* = 11) from 0 min to 90 min after administration of red wine without melatonin (PLC) or the same wine fortified with melatonin (MLT). Error bars represent the standard error of the mean (SEM). Statistical analysis: Different lower case or capital letters indicate differences within group and between groups, respectively (*p* ≤ 0.05, ANOVA, Tukey’s HSD test). (**D**) Time-course of the salivary melatonin mean levels in healthy volunteers (*n* = 11) from 0 min to 120 min after administration of red wine without melatonin (PLC) or the same wine fortified with melatonin (MLT). Error bars represent the standard error of the mean (SEM). Statistical analysis: Different lower case or capital letters indicate differences within group and between groups, respectively (*p* ≤ 0.05, ANOVA, Tukey’s HSD test).

After the administration of wines, a significant, steady decline of serum MLT was observed from baseline up to 90 min for both MLT+ and PLC wines, despite following different kinetics (Figure 2A–C). Patient No. 3, in particular, showed a strong peak of MLT at 60 min, after MLT+ wine intake (Figure 2A), not detectable after PLC (Figure 2B). The administration of MLT+ wine delayed the drop down of serum MLT producing a plateau at 30 and 60 min (8.6 ± 1.4 pg mL^−1^ and 8.7 ± 2.2 pg mL^−1^, respectively), no more detectable at 90 min (Figure 2C, ANOVA, *p* ≤ 0.05). Consistently, when MLT plasma concentrations obtained at different time points were corrected for individual baseline MLT concentrations, the *plateau* was confirmed. At 30 and 60 min, MLT+ wine produced a decrease of the indoleamine equal to −1.7 ± 1 pg mL^−1^ (11%) and −1.6 ± 2 pg mL^−1^ (14%), respectively, whilst PLC wine produced a decrease equal to −3.3 ± 1.3 pg mL^−1^ (20%) and −4.5 ± 1.3 pg mL^−1^ (37%).

The maximum concentration (C_max_) of MLT was 8.7 ± 2.2 pg mL^−1^, achieved 60 min (T_max_) after the intake of red wine enriched with this molecule, while, for PLC, C_max_ was 6.7 ± 0.6 pg mL^−1^ at 30 min (t_max_). The area under the curve (AUC) was 745 ± 88 pg min^−1^ per mL for PLC-wine and 993 ± 162 pg min^−1^ per mL for MLT+ wine. These differences were not statistically significant (two-sample *t*-test).

Salivary levels of MLT slightly peaked at 45 min after MLT+ wine intake, though without a statistical significance, returning to levels closer to the PLC at 120 min (Figure 2D).

### 2.2. Adverse Effects

Among side effects, sleepiness was the most frequently adverse event complained during the trial, more frequently after MLT+ wine intake (Table 2).

## 3. Discussion

In human, data on dietary MLT absorption are still scant, being currently documented just in two clinical trials [18,19]. Maldonado reported that, in seven healthy volunteers, serum MLT significantly increased 45 min after drinking beer containing MLT (total MLT ingested 112 ng for men and 56 ng for women) [19]. Sae-Teaw et al. [18] showed that, in 12 healthy male volunteers, serum MLT concentration peaked 120 min after fruit consumption (total MLT ingested 302 ng, 150 ng and 8.9 ng for pineapple, orange and banana, respectively).

To the best of our knowledge, to date, the effect of a glass of red wine intake on MLT levels has never been investigated, despite the overwhelming interest of research on this beverage and this molecule. Here, we aimed at clarifying the issue, by using a MLT-enriched wine containing the same amount of MLT naturally found in wine to reproduce, at the most, a real intake condition. We applied a highly accurate and reliable study design, i.e., a randomized, crossover, controlled clinical trial, which represents the highest level of evidence in clinical experimental research [20].

From our findings, although we could not distinguish unfailingly between endogenous and exogenous MLT and given the low dose contained in our wine and the sensitivity of LC/MS method applied as well, we found minor changes in serum MLT concentrations, as a reduced drop of the molecule over time. We can speculate that MLT+ wine may counteract the physiological decline of serum MLT during the morning. This physiological decline is consistent with previous literature reporting a decrease of serum MLT in volunteers from 10.98 pg mL^−1^ before 09.00 to 1.96 pg mL^−1^ after 11.00 [21]. The time points reflecting red wine MLT systemic absorption are consistent with the previous study on MLT in beer [19] and are slightly different from the ones reported by Sae-Teaw et al. [18] on MLT in fruits, which peaked at 120 min. The differences amongst the food matrixes could have affected the absorption rate: the presence of ethanol, as in our case, could have contributed, due to its solvent nature, to enhance the permeability of mucosal membranes of the gastrointestinal tract [22]. Moreover, previous studies [18,19] reported higher serum levels of MLT after food intake than ours. The different method used for serum analysis can explain, at least in part, these differences. In those studies [18,19], the MLT levels were determined by enzyme-linked immunosorbent (ELISA) assay. Here, we measured the serum MLT concentration in humans by the more accurate and reliable LC/MS technique, which overcomes the methodological issue related to ELISA cross-reactivity with unspecific molecules, such as MLT isomers and other indoleamines. Although this analytical approach is more precise than previously used ones, a further operational improvement would arise using LC-MS/MS technique allowing to detect MLT and respective metabolites more and more precisely. Besides the methodological issue, differences among studies can be ascribed to the MLT produced by the epithelial tissues of gastrointestinal tract and released within blood circulation, which can confound the real amount of dietary MLT absorbed [19,23]. The presence of putative and yet unidentified compounds (such as MLT precursors or phytochemicals) in the beer and fruits could also stimulate the biosynthesis of endogenous MLT, further complicating the picture [19].

On the other hand, we found a slight MLT peak in saliva after 45 min from MLT+ wine intake. We hypothesize that it could be due to the topical presence of wine MLT rather than coming from bloodstream. Food MLT, indeed, may bind the oral mucosa surface, similar to what occurs in presence of red wine polyphenols, although the precise mechanisms are still unknown [24]. Topical MLT may be in turn systemically absorbed via transmucosal absorption but at really very low level, as occurs with drugs [25]. MLT receptors are represented in the oral mucosa possibly to mediate specific biological activities [26]. In any case, we have to take into account that the topical presence of MLT can act as a confounder when assessing salivary levels of the molecule after food intake. This is of great relevance considering the low dose contained in wine and the even lower dose systemically absorbed after the ingestion.

The limitations of our study, however, impede conclusive dissertations. The major drawbacks include the small sample size and the need of verifying the presence, in serum and saliva, of red wine’s MLT isomers—possibly different from the endogenous ones. Moreover, future research should focus on considering the measurement of MLT 24-h rhythms that include nocturnal levels of the molecule, which can contribute to better elucidate how wine can affect individual’s circadian rhythm of endogenous MLT.

## 4. Materials and Methods

### 4.1. Volunteers

Participants included female and male young individuals (20–45 years of age), healthy, with normal weight (Body Mass Index, BMI, 18.5–25.0 kg m^−2^) who voluntarily accepted to join the study. All volunteers provided their written informed consent, signing a letter of agreement. For each individual, demographics, anthropometric characteristics and dietary habits were recorded, including consumption per day/week of alcoholic beverages, fruits, vegetables, tea and coffee; the level of physical activity (min per week); and the smoking habit. Among clinicians and students of the Departments involved in the study, individuals were considered eligible and were contacted. Exclusion criteria were: pregnancy and lactation for women; supplementation with MLT and/or any other dietary supplement (vitamins, antioxidants, botanicals, phytochemicals); systemic and chronic-degenerative diseases; abnormal hematological parameters; heavy smoking (more than 10 cigarettes/day) and heavy alcohol drinking (more than 8 standard drinks per week for women and more than 15 standards drinks per week for men); and high-intensity physical activity. None of the volunteers was taking any pharmacological treatment.

### 4.2. Ethical Approval

The study was conducted in accordance with the ethical principles of the Declaration of Helsinki and it received the ethics approval by the Institutional Ethical Committee (Università degli Studi di Milano, approved 10 July 2014).

### 4.3. Experimental Overview and Study Design

**Figure 3:**
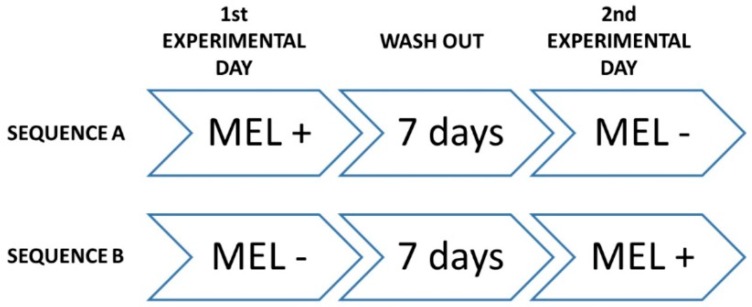
Sequential steps of study interventions.

The study was designed as triple-blind clinical trial, where participants, care providers, and investigators assessing outcomes where not informed on assignments to interventions. It was also randomized, crossover, placebo-controlled and registered at www.clinicaltrial.gov (ID: NCT02767102). All phases of the trial were performed at Clinica Odontoiatrica Università degli Studi di Milano, Polo San Paolo, where data were collected and analyzed (date range for participant recruitment: from 10 July 2014 to 17 July 2014; date range for performing the trial and follow-up: from 21 July 2014 to 31 July 2014). At baseline, demographics and anthropometric characteristics, dietary habits and physical conditions of the participants were recorded, and the inclusion/exclusion criteria were reviewed. Then, volunteers were randomized to receive one glass (125 mL) of red wine in two different experimental days, according to one of the following sequences (Figure 3): (i) sequence A—on first experimental day, the participant received placebo (PLC) red wine, followed by 1-week wash-out, and, on second experimental day, the same participant received MLT enriched (MLT+) red wine; and (ii) sequence B—on the first experimental day, the participant received MLT+ wine, followed by 1-week wash-out, and, on the second experimental day, the same participant received PLC wine.

A simple randomization method was applied. The randomization list was prepared using an online tool (http://graphpad.com/quickcalcs/randomise1.cfm) and allocation concealment was ensured because the person who generated the randomization list and assigned the volunteers to the two arms was not involved in evaluating eligibility of individuals and their enrolment. One of the authors (E.V.) administered the wine glass to volunteers, who were instructed to consume wine within 1–5 min, immediately after the first basal blood and saliva collection performed at 08:30. Both wines (with and without MLT) were administered in two identical glasses and they had the same color and flavor. Further blood withdrawals were collected at 30, 60 and 90 min, whereas further saliva samples were collected, via unstimulated spitting method [24], at 45 and 120 min after wine administration.

### 4.4. Dietary Control

A dietary control was performed the 24 h before the beginning of the trial and during the experimental day: individuals were required to avoid the consumption of alcoholic beverages, extra virgin olive oil, coffee, tea, nuts, rice, legumes, fruits, dairy products and mushrooms. Volunteers fasted overnight and came to the laboratory at 08:00, where they consumed a light MLT free breakfast (40 g of non-whole biscuits with 125 mL of warm water).

### 4.5. Placebo and MLT Enriched Red Wines

Placebo red wine (PLC wine) was a red wine naturally lacking of MLT, called Rosso di Marco (cv. Pignatello, Terre Siciliane IGT), which was formerly screened in our previous work on the MLT content in Sicilian red, white and dessert wines [9]. The farm De Bartoli (Pantelleria, Italy) kindly provided this wine. MLT enriched red wine (MLT+ wine) was the same red wine fortified with 10 ng mL^−1^ MLT: Briefly, 125 µL of an aqueous solution, containing MLT (*N*-Acetyl-5-methoxytryptamine, ≥99.5%, purchased from Sigma-Aldrich^®^, Saint Louis, MO, USA) at a concentration of 10 µg mL^−1^, was added to the wine to reach the final concentration. The enrichment corresponded to an intake of 1.25 µg of MLT per glass. This amount of MLT was selected because in agreement with the mean levels of MLT detectable in wines according to previous studies [6,7,8,27]. All analyses of wine MLT were carried out as already reported [9]. An investigator, not involved in the clinical trial and not aware of the randomization list, added the molecule. The two bottles, containing PLC or MLT+ red wines, were identical and just coded with 1 (MLT) or 2 (PLC). Only two members in the research team, M.I. and G.L., who did not participate in any of clinical phase or data collection or data analyses, were aware of the codes.

### 4.6. Melatonin Concentrations in Serum and Saliva

Serum MLT levels (primary outcome) were determined on blinded samples following the method described by Wang et al. [28] with slight modifications. We used a high performance liquid chromatography (Ultimate 3000, Dionex, Sunnyvale, CA, USA) coupled to a triple quadrupole mass spectrometer (ABSciex QTrap 3200, ABSciex, Milan, Italy) (LC-MS), using *N*-acetyltryptamine as internal standard. We preferred the solid-phase extraction instead of the liquid–liquid extraction with dichloromethane [28] to avoid the difficult transfer with a tip of an organic phase below the aqueous one. After pre-analytical processing, samples were injected in a ZORBAX Eclipse dsDNA Analysis (Agilent; Santa Clara, CA, USA) (2.1 mm i.d. × 75 mm, 3.5 µm) at 40 °C. The flow-rate was 0.4 mL min^−1^ and the samples were eluted by a gradient between (A) ammonium formate 2 mM with 0.1% formic acid, and (B) acetonitrile (30% B for 1 min, 30–60% B in 3 min, 60–99% B in 0.1 min, 99% B for 1 min, 99–30% B in 0.1 min, post run per 0.9 min). Total run time 6.2 min. The multiple reaction monitoring (MRM) technique was used for the quantification of MLT in serum samples, selecting the optimized fragmentation *m*/*z* 233 → 174, declustering potential (DP) 26 V and collision energy (CE) 20 V for MLT, and *m*/*z* 203 → 144, DP 15 V and CE 20 V for N-acetyltryptamine (internal standard). Salivary MLT was measured by ELISA kit (BTB-E1013Hu, Human Melatonin, MT ELISA Kit, Li StarFISH S.r.l.; Milan, Italy), following manufacturer instructions.

Maximum plasma concentrations (C_max_) and times to achieve maximum plasma concentrations (t_max_) were obtained directly by serum concentration time profile. The area under the plasma concentration–time curve (AUC) was determined according to the linear trapezoidal rule.

### 4.7. Volunteers’ Compliance and Adverse Effects

Volunteers consumed the glass of wine under the direct, visual supervision of the clinical investigator, who also recorded the exact time required to each participant to drink the glass of wine (from 1 to 5 min). During each experimental day, volunteers were instructed to record any side effect due to the wine drinking, using an ad hoc questionnaire. They were asked to indicate, in particular, loss of concentration, numbness, sleepiness and drowsiness, headache, gastric pain or any other adverse event.

### 4.8. Statistical Analysis

The sample size (*n* = 14) to assure at least the 80% statistical power was calculated, using an online statistical software (http://www.sample-size.net/means-effect-size/) and considering the 20% of dropouts. The calculation was based on the effect size and standard deviations observed from previous literature concerning MLT serum concentration after the intake of beverages containing MLT [18,19]. Statistical analysis was carried out on blinded data using OriginPro^®^ software (version 8.0, Northampton, MA, USA), and the results are represented as means with their standard errors (SEM). Data were analyzed using a per protocol approach. Within group and between groups comparisons were assessed by the two-way analysis of variance (ANOVA) for repeated measurements within participants. Significant within group and between groups differences were accepted at *p* ≤ 0.05 and comparison among means was determined according to the Tukey’s honestly significant difference (HSD) test. Two-sample *t*-test was calculated to assess differences of C_max_, t_max_ and AUC between groups.

## 5. Conclusions

Our results showed that, after the acute administration of a glass of MLT+ wine, levels of this indoleamine in the bloodstream change, peaking at 60 min after the intake. Noteworthy, the absorbed dietary MLT significantly counteracted the physiological decrease of the endogenous MLT in the serum. Salivary MLT slightly peaked at 45 min after MLT+ wine intake, probably because of a topical effect. Further studies are needed to elucidate the biological significance of dietary MLT in humans.

## Figures and Tables

**Table 1:** Demographics, anthropometric characteristics and dietary habits of participants (*n* = 12).

No.	Gender	Age (Years)	Height (cm)	Weight (Kg)	BMI (Kg m^−2^)	Fruits (Servings/Day)	Vegetables (Servings/Day)	Wine
1	M	22	171	65	22.2	0.5	0.5	<2 glasses/week
2	M	25	178	66	20.8	1	1	<2 glasses/week
3	M	43	179	89	27.8	0.5	0.5	2–7 glasses/week
4	F	24	164	52	19.3	2	2	1 glass/day
5	M	24	180	74	22.8	2	2	0
6	F	22	165	52	19	3	3	2–7 glasses/week
7	F	24	170	61	21	1	2	0
8	M	21	178	64	20.2	2	2	0
9	F	29	165	55	20.2	3	3	1 glass/day
10	F	23	157	48	19.5	1	1	0
11	M	24	178	76	24	2.5	0.5	0
12	M	29	185	74	21.6	0.5	0.5	1.5 glass/day
**Mean**		25.8	172.5	64.6	21.3	1.9	2	
**SD**		5.9	8.4	12	2.5	0.8	0.7	

**Table 2:** Side effects and adverse events reported by the volunteers (*n* = 11).

Wine	Loss of Concentration	Numbness	Sleepiness	Headache	Gastric Pain	Others
Melatonin	2	0	8	1	1	3 *
Placebo	2	1	6	1	2	0

* Flush, *n* = 2; tiredness, *n* = 1.

## Abbreviations

ELISA	enzyme-linked immunosorbent assay
LC-MS	liquid chromatography coupled to mass spectrometry
MLT	melatonin
PLC	placebo
